# Targeting the SOX11/GPX4/SLC7A11 Axis: A Novel Mechanism for Boric Acid-Induced Ferroptosis in Endometrial Cancer

**DOI:** 10.1007/s12011-026-05158-7

**Published:** 2026-05-25

**Authors:** Elif Karabacak, Ceyhan Hacioglu

**Affiliations:** 1 Faculty of Pharmacy, Department of Pharmacology, Düzce University, Düzce, Türkiye; 2 Faculty of Medicine, Department of Medical Biochemistry, Düzce University, Düzce, Türkiye

**Keywords:** Boric acid, Endometrial cancer, Ferroptosis, SOX11

## Abstract

Sex-determining region Y-related high-mobility group box 11 (SOX11) is recognized as a contributor to endometrial cancer (EC) progression, yet its specific involvement in ferroptosis regulation remains unclear. This research explores the potential of boric acid (BA) to influence both SOX11 expression and ferroptosis-related pathways within EC cells. Utilizing human EC-derived Ishikawa cells and non-malignant endometrial SHT290 cells, BA treatment regimens ranging from 100 µM to 25.6 mM were employed. Cellular viability and proliferative capacity were quantified using MTT and bromodeoxyuridine incorporation assays, respectively. Furthermore, quantitative reverse-transcription polymerase chain reaction (qRT-PCR) and western blot analyses were conducted to evaluate the expression profiles of *SOX11*, glutathione peroxidase 4 (*GPX4*), and solute carrier family 7 member 11 (*SLC7A11*). Ferroptosis was evaluated through measurements of malondialdehyde (MDA), glutathione (GSH), Fe²⁺ content, and lipid peroxidation using the C11-BODIPY581/591 probe. BA reduced cell viability and proliferation in a time- and concentration-dependent manner, with IC₅₀ values of 2.6 mM and 15.5 mM in Ishikawa and SHT290 cells, respectively. In Ishikawa cells, BA induced detectable lipid peroxidation, cytomorphological changes, elevated MDA and Fe²⁺ levels, and depleted GSH content—hallmarks of ferroptosis. BA treatment decreased *GPX4* and *SLC7A11* expression while significantly downregulating *SOX11* at both transcript and protein levels. These findings indicate that BA promotes ferroptosis and inhibits proliferation in EC cells, at least in part through suppression of *SOX11* and downregulation of the GPX4/SLC7A11 axis. This study reveals a previously unreported regulatory mechanism and indicates that BA merits further investigation as a potential therapeutic agent targeting ferroptosis in EC.

**Supplementary Information:**

The online version contains supplementary material available at 10.1007/s12011-026-05158-7.

## Introduction

Globally, endometrial carcinoma (EC) represents a significant health concern, ranking as the sixth most prevalent cancer in women and the fourteenth most common cancer overall [1]. In the United States, according to 2016 American Cancer Society statistics, this malignancy accounted for approximately 60,000 new diagnoses and an estimated 10,000 mortalities [2]. Notably, a significant subset of patients (2–14%) comprises premenopausal women under 40 years of age who prioritize fertility preservation [3]. Globally, epidemiological reports from 2018 identified over 380,000 incident cases of EC, with endometrial adenocarcinoma—the predominant histological subtype—accounting for roughly 85% of these occurrences [4]. The pathogenesis of EC is principally driven by uncontrolled proliferation of endometrial epithelial cells [5]. Although chemotherapeutic agents remain a cornerstone of systemic treatment for advanced or recurrent endometrial malignancies, their clinical utility is constrained by substantial toxicities that adversely impact patient quality of life. This ongoing challenge necessitates the development of targeted therapeutic approaches that can selectively eliminate malignant cells while preserving healthy tissues, thereby optimizing both oncological outcomes and patient well-being.

The SOX gene family, encompassing 20 Sry-related HMG box transcription factors in humans, includes the highly conserved SOXC subgroup (SOX4, SOX11, SOX12), where functional redundancy is suggested by evolutionary conservation [6]. SOX11 critically mediates the neoplastic progression of ductal carcinoma in situ (DCIS) to metastatic invasive breast cancer, establishing its fundamental role in cancer pathology [7]. This oncogenic factor exhibits a distinct expression ontogeny: undetectable in normal adult breast epithelial cells but prominent during mammary development (embryonic epithelium [8], fetal stem cells [9]) and pathologically re-expressed in basal-like invasive carcinomas [10]. Mechanistically, aberrant SOX11 upregulation in Brca1-deficient mouse models drives aggressive phenotypes characterized by local invasion and distant dissemination to osseous, pulmonary, and cerebral sites [11].

Ferroptosis, an iron-dependent, non-apoptotic regulated cell death pathway initiated by cystine depletion and lipid peroxidation with reactive oxygen species (ROS) involvement [12], offers significant promise as a novel cancer treatment modality due to its tumor-selective effects [13]. Molecular control of this cascade involves a critical network, notably featuring solute carrier family 7 member 11 (SLC7A11), which mediates cellular cystine import necessary for glutathione (GSH) production [14]. A further pivotal inhibitor is glutathione peroxidase 4 (GPX4), which requires reduced GSH as a cofactor to neutralize cytotoxic lipid hydroperoxides and thereby inhibit ferroptosis execution [15]. Recent investigations demonstrate that SOX4 knockdown in osteosarcoma cells induces p53 and pro-apoptotic Bax upregulation while downregulating anti-apoptotic Bcl-2 [16]. Transcriptional profiling studies in ovarian malignancies reveal elevated SOX11 mRNA expression in epithelial subtypes, correlating with improved disease-specific survival and reduced recurrence rates [17]. Notably, a previous study has demonstrated that SOX11 promoter methylation is significantly more prevalent in malignant endometrial tissues compared to normal endometrium, correlating with decreased gene expression and more aggressive clinicopathological features [18]. However, the pathophysiological relevance of this SOX11-ferroptosis regulatory axis in EC remains undetermined, particularly its potential role in modulating ferroptosis during neoplastic progression.

Boron, an essential micronutrient in human physiology, is predominantly obtained through dietary intake of plant-derived boron compounds and from aqueous boric acid (BA) solutions [19]. Biochemical analyses indicate that circulating boron in human plasma maintains a physiological concentration range of 10–20 µM, with BA representing its principal bioavailable form [20]. Preclinical investigations across multiple cancer models, including prostate adenocarcinoma, glioblastoma, and hepatocellular carcinoma, reveal that boron-based compounds exert antiproliferative effects through mechanisms including apoptosis induction and cell cycle arrest [21–23]. While the precise molecular mechanisms underlying boron’s anticancer activity remain incompletely characterized, its pleiotropic biological activities continue to generate sustained scientific interest, particularly in the context of oncotherapeutic development and multifactorial disease modulation.

This study was designed to investigate the mechanistic role of SOX11 in regulating tumor cell proliferation following BA exposure, while concurrently interrogating its regulatory influence on ferroptotic pathways and EC progression. The experimental framework specifically evaluated BA’s therapeutic potential in Ishikawa EC cell models, with emphasis on its capacity to modulate the SOX11/GPx4/SLC7A11 signaling axis—a critical pathway governing redox homeostasis and iron-dependent cell death mechanisms.

## Materials and Methods

### Cell Culture and Treatments

To assess the anticancer potential of BA, experiments were performed on two human endometrial cell lines: Ishikawa cells, derived from EC, and SHT290 cells, representing normal endometrial stroma, both obtained from the American Type Culture Collection (ATCC). Cells were maintained in a humidified 5% CO₂ incubator at 37 °C in DMEM basal medium supplemented with 10% (v/v) FBS and dual antibiotics: penicillin (100 units/mL) and streptomycin (100 µg/mL). To assess BA efficacy, cellular responses to a concentration range spanning 100 µM to 25.6 mM [24] were measured following 24 h and 48 h exposure periods for both cell types.

### Cell Viability Assessment

Cytotoxicity assessment of Ishikawa and SHT290 cell lines was performed using the MTT assay. Cells were seeded into 96-well plates at an initial density of 5 × 10³ cells per well, suspended in 100 µL of complete growth medium, and incubated overnight to facilitate adherence. Following attachment, the cells were exposed to a concentration gradient of BA (100 µM to 25.6 mM), solubilized in dimethyl sulfoxide (DMSO; Sigma-Aldrich, cat no: 472301), for treatment durations of 24 h and 48 h. Subsequent to treatment, the medium was aspirated and replaced with 100 µL of fresh medium containing MTT at a final concentration of 5 mg/mL. A further 4-hour incubation period enabled intracellular formazan crystal formation. The MTT solution was then carefully removed, and 100 µL of DMSO was introduced into each well to solubilize the intracellular formazan deposits. Absorbance quantification was conducted at a wavelength of 570 nm utilizing a BioTek microplate reader, with each experimental condition analyzed across eight technical replicates.

### Cell Proliferation via BrdU Assay

Cell proliferation was further assessed employing the BA concentration eliciting significant cytotoxicity, as empirically determined by MTT dose-response profiling. To assess proliferative modulation, Ishikawa and SHT290 cells were plated in 6-well plates at an identical density of 2 × 10⁵ cells per well and subjected to BA treatment for 24 h. DNA replication dynamics during the S-phase were evaluated via a 5-bromo-2′-deoxyuridine (BrdU) incorporation assay (Sigma-Aldrich, 2750), wherein BrdU integration into nascent DNA strands was immunodetected using anti-BrdU monoclonal antibodies. Quantitative analysis of proliferation rates was performed by measuring 450 nm values on a microplate reader, adhering strictly to the manufacturer’s analytical guidelines.

### Quantification of Intracellular Biomarkers

The levels of GSH, malondialdehyde (MDA), and ferrous iron (Fe²⁺) were measured using commercially available ELISA kits (catalog numbers: MBS727656, MAK085, and MAK025, respectively), each utilizing pre-coated antigen-specific antibodies. Following 24 h exposure to BA, cells were washed with ice-cold phosphate-buffered saline (PBS; pH 7.0). Subsequent harvesting involved centrifugation at 1,500 × *g* for 10 min (4 °C), yielding pellets that underwent resuspension in lysis buffer followed by 60 s mechanical homogenization. The homogenates were then clarified by a second centrifugation step (1,500 × *g*, 5 min, 4 °C) to eliminate debris. Supernatants (cell lysates) were aliquoted and stored at − 20 °C until further use. Quantitative analysis of the target molecules was conducted via competitive ELISA, following the manufacturers’ recommended protocols. Color development, corresponding to antigen–antibody binding, was measured at 450 nm using a microplate reader.

### Lipid Peroxidation Assay

To assess lipid peroxidation as an indicator of ferroptosis, the fluorescent probe C11 BODIPY581/591 (Invitrogen) was employed. A 10 mM stock solution was prepared by reconstituting 1 mg of the compound in 0.2 mL dimethyl sulfoxide (DMSO) and preserved at − 20 °C under light-protected conditions. Immediately prior to cell staining, this stock was diluted in complete culture medium to achieve a 10 µM working concentration. Cells, cultured under standard conditions (37 °C, 5% CO₂) in 6-well plates and subjected to designated experimental treatments, were incubated with the 10 µM C11 BODIPY581/591 probe in darkness for 30 min to facilitate uptake. Following triple washing with sterile phosphate-buffered saline (PBS) to eliminate unincorporated probe, lipid peroxidation levels were evaluated via fluorescence imaging using an Olympus inverted microscope (20× objective). For each experimental condition, 50 randomly selected fields were captured. Quantitative analysis of lipid peroxide production was conducted by measuring the mean fluorescence intensity in the captured images using ImageJ software. Briefly, regions of interest were drawn around individual cells, and the integrated density was measured after background subtraction. The results are presented as a fold-change increase in the BA-treated groups relative to control conditions. For morphological assessment, phase-contrast images were captured at 20× magnification.

### Western Blot Analysis

Protein analysis commenced with cell lysis in RIPA buffer + inhibitors following 24 h BA treatment. Following centrifugation (12,000 × g, 15 min, 4 °C) to clarify cellular lysates, total protein concentration was determined spectrophotometrically using the BCA assay (Millipore, 71285), calibrated against appropriate standard curves. Aliquots representing 20 µg of heat-denatured protein per sample underwent electrophoretic separation on 10% SDS-polyacrylamide gels under reducing conditions. Resolved proteins were subsequently transferred onto nitrocellulose membranes employing a semi-dry transfer apparatus. To abrogate non-specific interactions, membranes were blocked for 1 h at ambient temperature using Tris-buffered saline containing 0.1% Tween-20 (TBST) supplemented with 2% (w/v) bovine serum albumin (BSA). Immunological detection commenced with an overnight incubation at 4 °C utilizing the following primary antibodies: anti-GPX4 (PA5-102521; 1:1,000), anti-SLC7A11 (PA1-16893; 1:1,000), anti-SOX11 (MA5-32395; 1:1,000), and anti-β-actin (PA1-183; 1:2,000). After three rigorous washes with TBST, membranes were incubated for 1 h at room temperature with corresponding horseradish peroxidase (HRP)-conjugated secondary antibodies (1:5,000). Antigen-antibody complexes were visualized via enhanced chemiluminescence (ECL) using a Bio-Rad imaging system, with relative protein expression levels quantified by densitometric analysis of band intensities.

### Quantitative Reverse-Transcription Polymerase Chain Reaction (qRT-PCR) Analysis

Cells were seeded into six-well plates at a density of 5 × 10³ cells per well, and permitted to adhere. Following adherence, the dose groups received treatment with BA at designated concentrations for 24 h. Subsequent to treatment, the culture medium was aspirated and cellular RNA was isolated by direct lysis using 500 µl of Trizol reagent per well. Total RNA was purified according to standard procedures. Complementary DNA (cDNA) synthesis was subsequently performed utilizing a commercial cDNA synthesis kit, adhering strictly to the manufacturer’s protocol. Relative quantification of mRNA expression levels was conducted via qRT-PCR employing SYBR Green chemistry. The β-actin gene served as the endogenous reference control for normalization. The nucleotide sequences of the oligonucleotide primers used for qRT-PCR amplification were as follows: *GPX4* forward 5′-CGA GCT AAG GCA ATG CCA TGC-3′, *GPX4* reverse 5′-GAC GTG GCG CTT GGT CCA GT-3′, *SLC7A11* forward 5′-GGC TGG GAT TGA TTG GCC GCT-3′, *SLC7A11* reverse 5′-GAC TGC TTG AGT CCC GCA-3′, *SOX11* forward 5′-GCA TGA CAC GTC AGC TGG TCA-3′, *SOX11* reverse 5′-CGA GAC GAT GCC CTC AAG GCT-3′, *β-actin* forward 5′-GCC TCA AGG CTT CTG AGT CAG-3′, *β-actin* reverse 5′-GGC TCG AAA TGT TGC TCC GTG-3′.

### *SOX11* siRNA Transfection

Cells were seeded in the appropriate culture plates and allowed to reach approximately 60% confluence prior to transfection. Transient knockdown of SOX11 expression was achieved using small interfering RNA (siRNA) delivered via Lipofectamine™ RNAiMAX (Thermo Fisher Scientific), in accordance with the manufacturer’s protocol. Briefly, cells were transfected with 50 nM *SOX11*-targeting siRNA diluted in Opti-MEM reduced-serum medium. For the control group, cells were transfected with 50 nM scrambled non-targeting siRNA (Santa Cruz Biotechnology, sc-37007) using the same Lipofectamine™ RNAiMAX and Opti-MEM reduced-serum medium conditions. All other experimental conditions, including cell seeding density, incubation time (6 h), and post-transfection recovery period (24 h), were identical between the *SOX11* siRNA and scrambled control groups. The siRNA duplex targeting human *SOX11* was designed as follows: sense: 5′-GGAGCUGGAGAUGAAGAAAUU-3′; antisense: 5′-UUUCUUCAUCUCCAGCUCCUU-3′.

### Statistical Analysis

All statistical analyses were conducted employing GraphPad Prism software (version 8.0). Data are expressed as mean values ± standard deviation (SD). Assessment of normal distribution within individual experimental groups was performed using the Shapiro-Wilk test, with a predefined significance level of *p* < 0.05. For experimental designs comprising multiple groups, determination of statistical significance was achieved through one-way analysis of variance (ANOVA). Where ANOVA indicated significant differences, subsequent pairwise comparisons were executed utilizing Tukey’s honestly significant difference (HSD) post-hoc test.

## Results

### Cellular Viability and Proliferative Capacity in Both SHT290 and Ishikawa Cell Lines were Modulated by BA Exposure

**Fig. 1:**
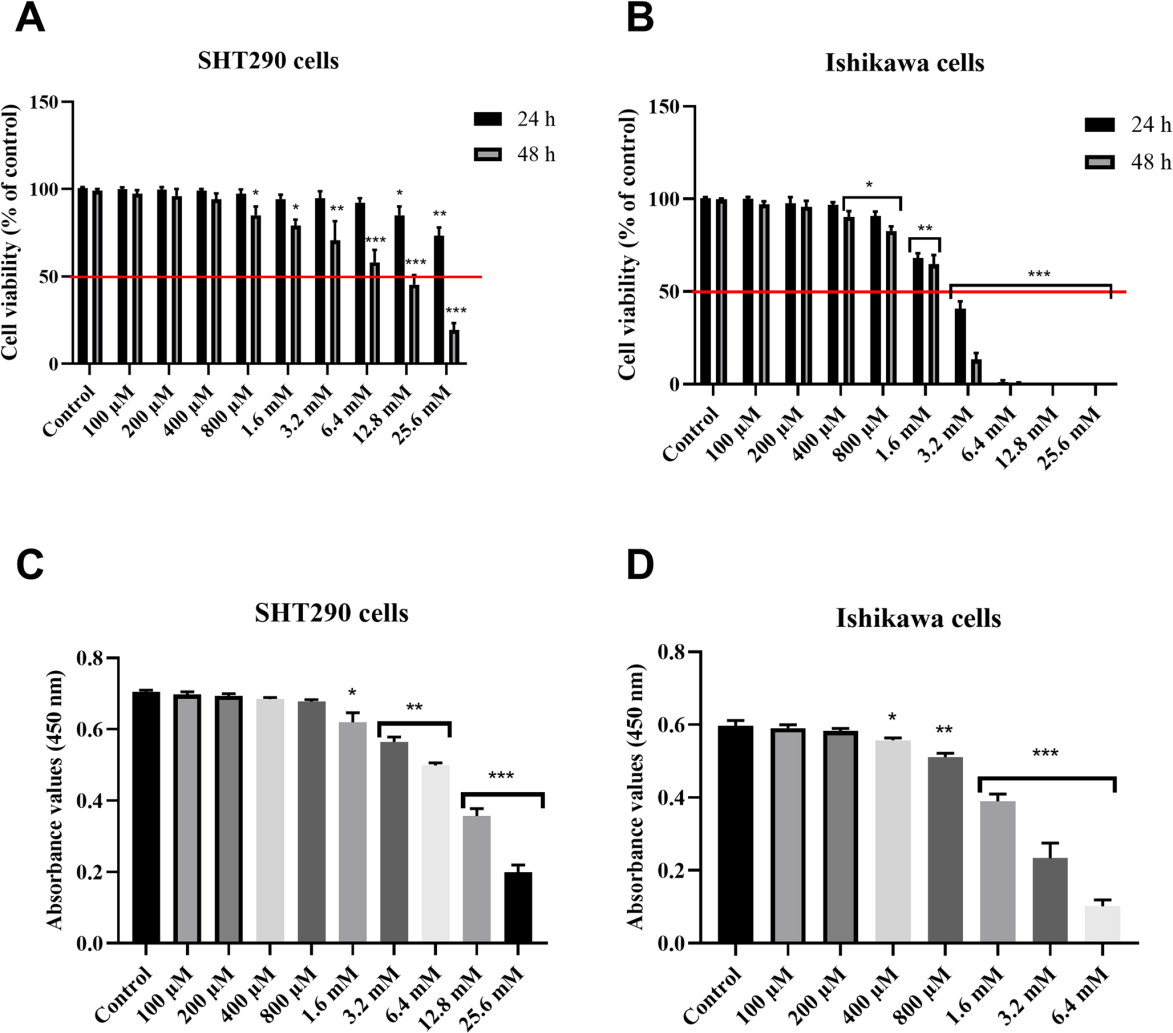
Boric acid-mediated suppression of cell viability and proliferation in SHT290 and Ishikawa cell lines. Cellular viability, quantified via MTT assay, was assessed for SHT290 cells in Fig. 1**A** and for Ishikawa cells in Fig. **1B**, **C **and **D** display proliferation rates, determined by BrdU assay, in SHT290 and Ishikawa cells, respectively. Data were presented as mean ± SD from seven independent experiments (*n* = 7). Significant differences compared to control groups are indicated as follows: *p < 0.05, **p < 0.001, ***p < 0.0001

BA exhibited differential cytotoxic thresholds between endometrial cell lines (Fig. 1A and B). MTT analysis of SHT290 cells treated with BA (100 µM–25.6 mM) revealed concentration- and time-dependent cytotoxicity (Fig. 1A). Viability remained statistically unaltered (*p* > 0.05) at concentrations ≤ 6.4 mM across both 24 h and 48 h exposures. Significant reductions emerged at ≥ 12.8 mM, with 24 h viability declining by 13.8% (12.8 mM; *p* < 0.01) and 30.6% (25.6 mM; *p* < 0.0001). After 48 h, progressive cytotoxicity manifested: 8.3% reduction at 800 µM, escalating to 89.4% at 25.6 mM (all *p* < 0.0001).

Ishikawa cells exhibited heightened sensitivity (Fig. 1B). At 24 h, concentrations ≤ 400 µM showed no significant viability loss (*p* > 0.05), whereas 800 µM–6.4 mM induced reductions from 10.4% to 98.7% (*p* < 0.05 to *p* < 0.0001). By 48 h, cytotoxicity initiated at 400 µM (9.3% reduction; *p* < 0.0001), intensifying to near-complete ablation (99.4%) at 6.4 mM. Notably, cell viability was entirely abolished at 12.8 mM and 25.6 mM after 24 h and 48 h.

The MTT assay data revealed a marked difference in cellular sensitivity, with Ishikawa cells exhibiting significantly greater susceptibility to BA’s inhibitory effects compared to SHT290 cells. This differential cytotoxicity was quantified by 24 h IC50 values of 2.6 mM for Ishikawa cells and 15.5 mM for SHT290 cells.

Cell proliferation, evaluated via BrdU incorporation assay after a 24 h BA treatment, revealed distinct responses between cell lines. Exposure to BA across concentrations of 100 µM to 800 µM elicited no statistically significant change in SHT290 cell proliferation relative to untreated controls (*p* > 0.05; Fig. 1C). Conversely, a pronounced anti-proliferative effect was observed at higher BA concentrations (1.6 mM, 3.2 mM, 6.4 mM, 12.8 mM, 25.6 mM), manifesting as significant inhibition rates of 7.3%, 21.9%, 36.8%, 60.1%, and 84.1%, respectively (*p* < 0.05, *p* < 0.001, *p* < 0.0001 versus control). Ishikawa cells, however, displayed markedly enhanced susceptibility to BA. Whereas concentrations ≤ 200 µM produced no significant proliferative alteration, substantial suppression occurred at 400 µM, 800 µM, 1.6 mM, 3.2 mM, and 6.4 mM BA, yielding 24 h inhibition rates of 10.5%, 18.1%, 39.4%, 78.5%, and 96.1%, respectively (*p* < 0.05, *p* < 0.001, *p* < 0.0001 versus control; Fig. 1D). These findings demonstrate a cell type-dependent anti-proliferative response to BA, with Ishikawa cells showing heightened sensitivity relative to SHT290 cells. This differential susceptibility highlights the critical role of cell-specific determinants in assessing BA’s therapeutic utility. Consequently, subsequent mechanistic analyses investigating BA’s effects on SOX11/GPx4/SLC7A11 signaling pathways were conducted using the more responsive Ishikawa cell line.

### BA Triggers Ferroptotic Cell Death in Ishikawa Cells via Lipid Peroxidation, GSH Depletion, and Iron Dysregulation

**Fig. 2:**
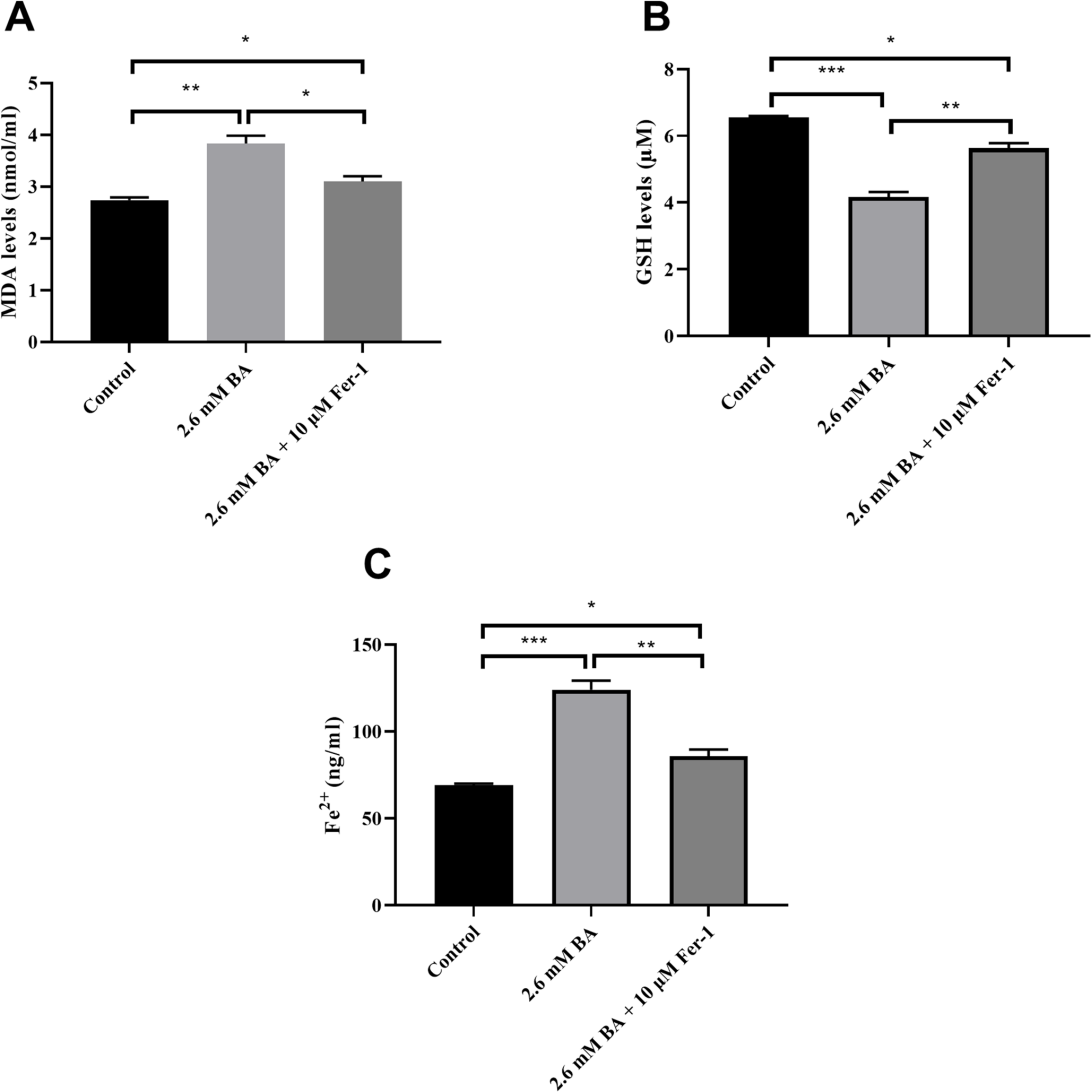
Impact of boric acid on lipid peroxidation, antioxidant status, and iron homeostasis in ishikawa cells. (**A**) MDA levels, (**B**) GSH levels, (**C**) Fe²⁺ levels. Data were presented as mean ± SD from seven independent experiments (*n* = 7). *p < 0.05, **p < 0.001, and ***p < 0.0001

Experimental findings obtained through ELISA demonstrated that 2.6 mM BA induced oxidative dysregulation in Ishikawa cells. To explore whether these alterations are linked to ferroptotic cell death, cells were initially incubated with 10 µM Ferrostatin-1 (Fer-1), a ferroptosis-specific inhibitor, for six hours before being subjected to BA for 24 h. This pre-treatment reduced BA-driven biochemical shifts. Following BA exposure, there was a marked elevation in MDA levels—an oxidative degradation product of lipids—and a significant reduction in the cellular antioxidant GSH (*p* < 0.0001 for both, Fig. 2A and B). Concomitantly, intracellular Fe²⁺ accumulation was significantly enhanced (Figs. 2C). However, these pro-oxidative shifts were notably reversed by Fer-1 pretreatment, which restored GSH content and suppressed the increases in MDA and Fe²⁺. These data demonstrate that BA treatment induces oxidative disturbances and biochemical changes consistent with ferroptosis.

### BA Treatment Induced Alterations in Cytostructural Integrity and Lipid Peroxidation Index

**Fig. 3:**
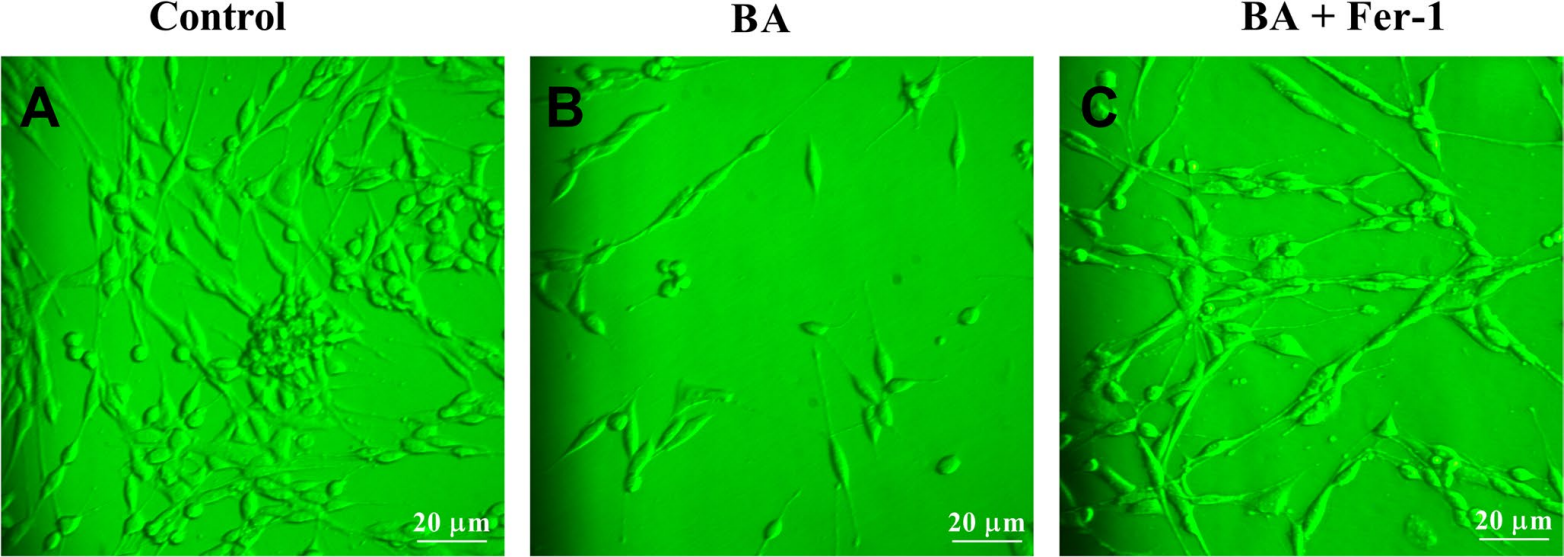
Fer-1 Attenuates BA-induced morphological aberrations and ferroptosis in ishikawa Cells. (**A**-**C**) Representative inverted microscopy images (20× objective): Control (**A**), BA-treated (**B**), BA + Fer-1-treated (**C**). Scale bar = 20 μm

Microscopic evaluation using inverted microscopy demonstrated a decline in cellular density within Ishikawa cell cultures following 2.6 mM BA exposure (Fig. 3A-C). Concomitantly, progressive morphological alterations were observed, characterized by diminished cell size and a marked reduction in cytoplasmic content. These morphological perturbations exhibited increasing severity in direct correlation with treated BA concentration. However, these BA-induced morphological damages were significantly mitigated by Fer-1 pretreatment, which restored normal cell size and preserved cytoplasmic integrity. These data collectively imply that BA promotes morphological disturbances in Ishikawa cells, most likely by activating ferroptosis-associated pathways.

**Fig. 4:**
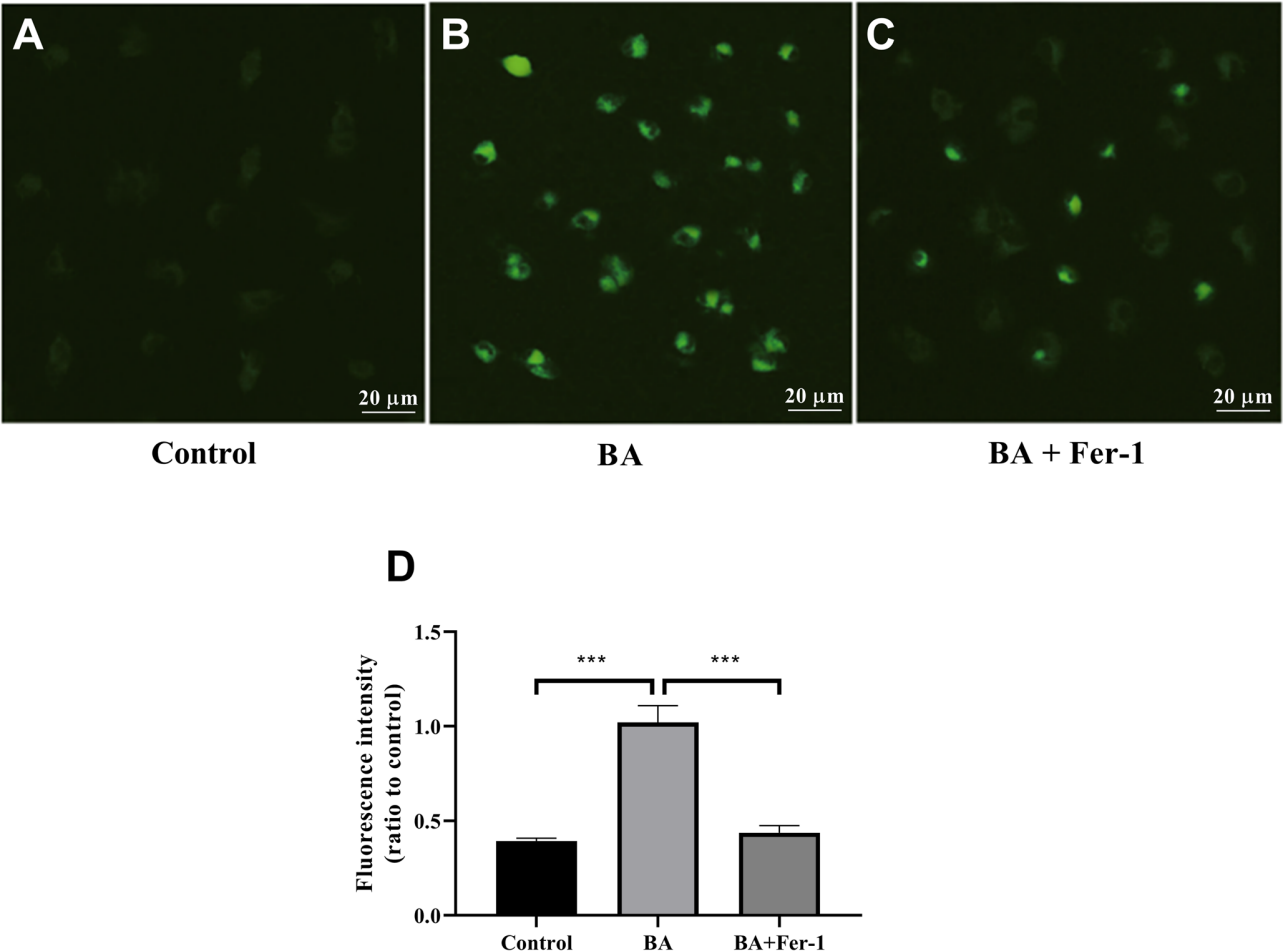
Ferroptosis inhibitor Fer-1 ameliorated BA-induced lipid peroxidation in Ishikawa cells. (**A**-**C**) Representative C11-BODIPY fluorescence images of lipid ROS. Control (**A**), BA-treated (**B**), BA + Fer-1-treated (**C**), Fluorescence intensity (**D**). Scale bar = 20 μm. Data were presented as mean ± SD from seven independent experiments (*n* = 7). ***p < 0.0001

BA exposure significantly increased lipid peroxidation in Ishikawa cells, as indicated by elevated fluorescence intensity of the lipid peroxide-sensitive C11-BODIPY581/591 probe. Quantitative analysis of lipid peroxidation using this probe after a 24-hour treatment with 2.6 mM BA revealed substantial cell death (Fig. 4A–D). Compared to untreated controls, a significantly greater fraction of cells treated with BA displayed lipid peroxide accumulation (*p* < 0.05). The lipid peroxidation index, determined from fluorescence measurements in 10 randomly selected microscopic fields at 20× magnification, confirmed a marked increase induced by BA treatment. Importantly, this elevation was nullified upon pretreatment with the ferroptosis inhibitor Fer-1, as no statistically significant difference was observed between the Fer-1 + BA group and the control group (*p* > 0.05). Taken together, these findings demonstrate that BA significantly increases lipid peroxidation in Ishikawa cells, predominantly through activation of ferroptosis pathways, as evidenced by the effective suppression of this response by Fer-1.

### BA Induces Ferroptosis via *SOX11* Suppression in Ishikawa Cells

**Fig. 5:**
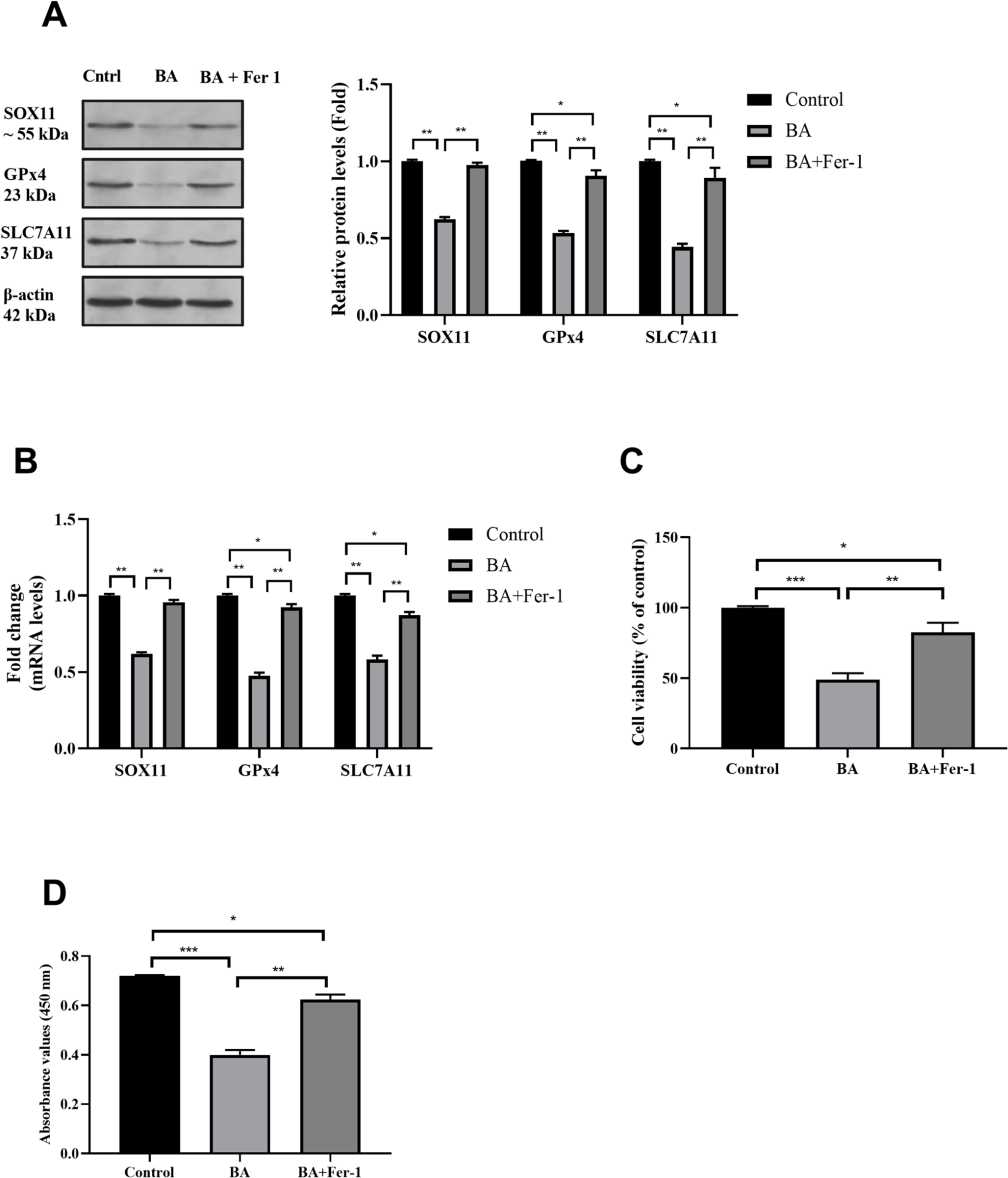
Regulation of the SOX11/GPX4/SLC7A11 Axis by BA and Fer-1 in Ishikawa Cells. (**A**) Western blot analysis of SOX11, GPX4, and SLC7A11 proteins. (**B**) qRT-PCR analysis of *SOX11*, *GPX4*, and *SLC7A11* transcript levels. (**C**) MTT results in Ishikawa cells. (**D**) BrdU incorporation in Ishikawa cells. Data were presented as mean ± SD from seven independent experiments (*n* = 7). Representative cropped blots are shown in Fig. 5A; the corresponding full-length uncropped blots are presented in Supplementary Figure S1. *p < 0.05, **p < 0.001 and ***p < 0.0001

To investigate BA’s functional interplay with *SOX11* within ferroptosis pathways, Ishikawa cells underwent 10 µM Fer-1 pretreatment for 6 h prior to 24-hour 2.6 mM BA exposure. Subsequent analysis demonstrated marked BA-induced suppression of *SOX11* expression, with protein and mRNA levels reduced by 47.2% and 40.9%, respectively, versus untreated controls (*p* < 0.001; Fig. 5A and B). Crucially, Fer-1 co-treatment substantially rescued this downregulation, restoring SOX11 protein to 70.4% and mRNA to 62.5% of baseline values compared to BA-treated group (*p* < 0.001), with resulting expression levels in the Fer-1 + BA group exhibiting no significant difference from untreated controls.

Parallel reductions in ferroptosis-related biomarkers *GPX4* and *SLC7A11* were observed following BA exposure. Ishikawa cells exhibited a 64.6% decrease in GPX4 protein and 51.8% reduction in mRNA (*p* < 0.001), alongside concomitant declines of 68.5% in SLC7A11 protein and 63.1% in mRNA expression versus controls (*p* < 0.001; Fig. 5A and B). Fer-1 treatment effectively abolished these suppressive effects, restoring both *GPX4* and *SLC7A11* expression. Notably, the high basal SOX11 expression characteristic of Ishikawa cells similarly diminished under BA-mediated ferroptotic conditions.

Exposure to BA significantly impaired the viability and proliferative potential of Ishikawa cells, as quantified by MTT and BrdU incorporation assays (Fig. 5C and D). This cytotoxic and anti-proliferative response, however, was effectively abrogated by pretreatment with the ferroptosis inhibitor Fer-1, which substantially restored cellular viability and proliferation. Collectively, these findings indicate that ferroptosis-related pathways mediate the observed effects for BA-induced disruption of redox homeostasis in this cellular model.

### *SOX11* Silencing Enhances BA-Induced Ferroptotic Biomarker Alterations

**Fig. 6:**
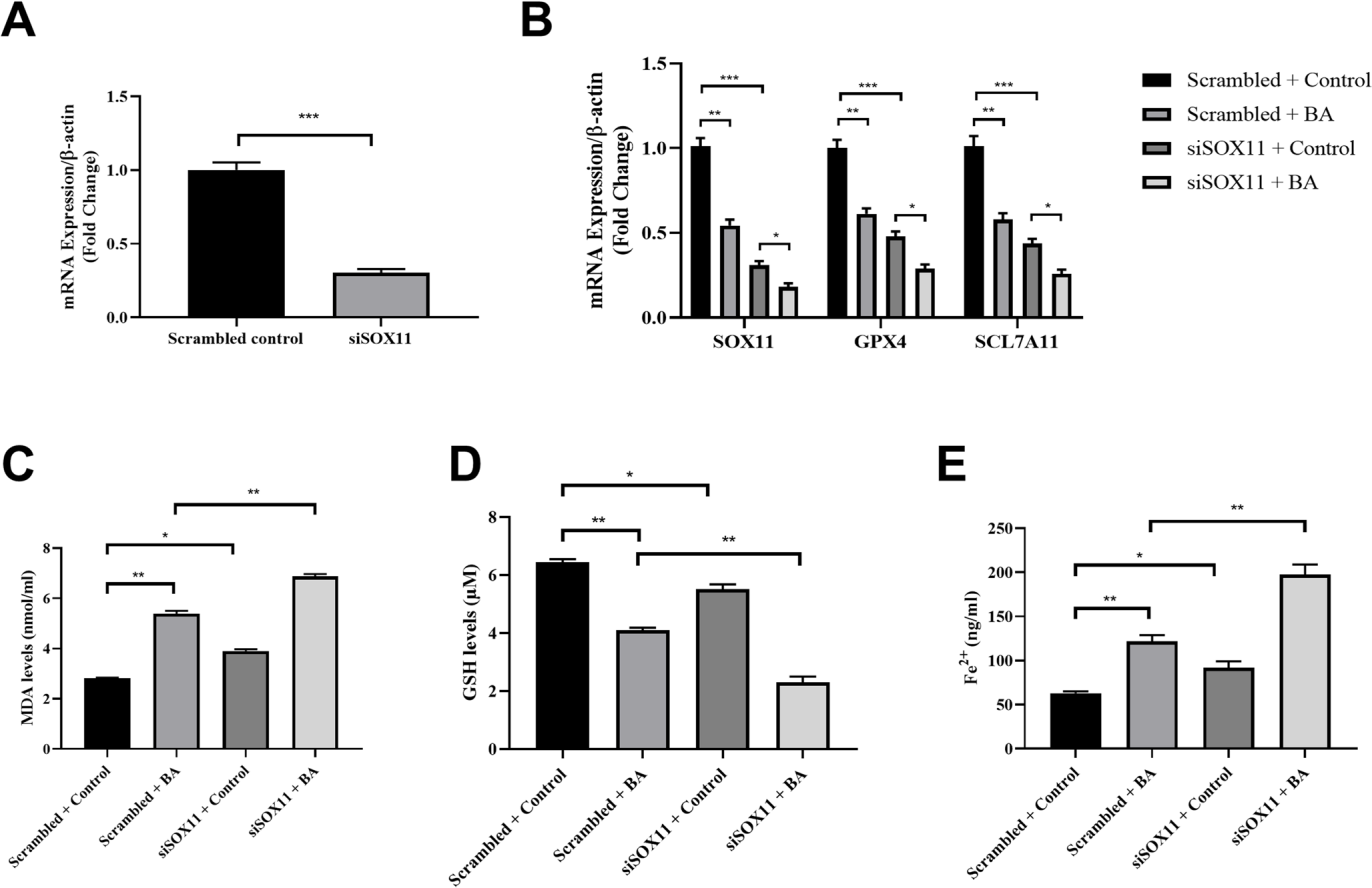
*SOX11* silencing enhances boric acid-induced ferroptotic responses in Ishikawa cells. (**A**–**B**) qRT-PCR analysis of *SOX11*,* GPX4*, and *SLC7A11* mRNA expression levels in cells transfected with scrambled control or siSOX11 and treated with control or BA. SOX11 knockdown efficiency was confirmed by a significant reduction in *SOX11* expression. (**C**–**E**) ELISA-based quantification of ferroptosis-associated biochemical markers, including MDA, GSH, and Fe²⁺. Data were presented as mean ± SD from seven independent experiments (*n* = 7). *p < 0.05, **p < 0.001, ***p < 0.001

To evaluate the functional role of *SOX11* in BA-mediated ferroptosis, *SOX11* expression was transiently silenced using siRNA in Ishikawa cells. Twenty-four hours post-transfection, quantitative qRT-PCR analysis revealed a significant reduction in SOX11 mRNA levels in siSOX11-transfected cells compared to scrambled control cells (0.31 ± 0.05 vs. 1.00 ± 0.08, respectively; *p* < 0.0001; Fig. 6A), confirming efficient gene knockdown.

To further investigate the interaction between *SOX11* suppression and BA treatment, cells were divided into four experimental groups: Scrambled + Control, Scrambled + BA, siSOX11 + Control, and siSOX11 + BA (Fig. 6B). Basal *SOX11* expression was highest in the Scrambled + Control group (1.00 ± 0.08), while BA treatment alone significantly reduced *SOX11* levels in Scrambled + BA cells (0.54 ± 0.06; *p* < 0.001). SOX11 knockdown alone (siSOX11 + Control) resulted in a more pronounced decrease (0.31 ± 0.05; *p* < 0.0001 vs. Scrambled + Control). Notably, the combination of *SOX11* silencing and BA treatment (siSOX11 + BA) led to the lowest *SOX11* expression levels (0.18 ± 0.04; *p* < 0.01 vs. Scrambled + BA), suggesting an additive suppressive effect.

Consistent with the regulatory role of *SOX11* in ferroptosis, the expression levels of key ferroptosis-associated genes, *GPX4* and *SLC7A11*, were also assessed by qRT-PCR. *GPX4* expression was highest in Scrambled + Control cells (1.00 ± 0.07), decreased following BA treatment (0.61 ± 0.05; *p* < 0.001), and was further reduced upon *SOX11* silencing (siSOX11 + Control: 0.48 ± 0.06; *p* < 0.0001). The most substantial suppression was observed in the siSOX11 + BA group (0.29 ± 0.05; *p* < 0.01 vs. Scrambled + BA). A similar trend was observed for *SLC7A11* expression, which declined from 1.00 ± 0.09 in Scrambled + Control cells to 0.58 ± 0.06 in Scrambled + BA cells (*p* < 0.001), 0.44 ± 0.05 in siSOX11 + Control cells (*p* < 0.0001), and reached the lowest level in siSOX11 + BA cells (0.26 ± 0.04; *p* < 0.01 vs. Scrambled + BA). These findings indicate that *SOX11* positively regulates *GPX4* and *SLC7A11* expression, and its suppression enhances BA-induced inhibition of ferroptosis defense mechanisms.

MDA levels, indicative of lipid peroxidation, were lowest in Scrambled + Control cells (2.92 ± 0.15 nmol/ml) and significantly increased upon BA treatment (5.49 ± 0.21; *p* < 0.001; Fig. 6C). *SOX11* silencing alone moderately elevated MDA levels (siSOX11 + Control: 3.68 ± 0.16; *p* < 0.05), while the combined treatment (siSOX11 + BA) resulted in the highest MDA levels (6.76 ± 0.24; *p* < 0.001 vs. Scrambled + BA), indicating enhanced lipid peroxidation.

In contrast, intracellular GSH levels showed an inverse pattern. GSH content was highest in Scrambled + Control cells (6.63 ± 0.19 µM) and significantly decreased following BA treatment (4.11 ± 0.15; *p* < 0.001; Fig. 6D). *SOX11* knockdown alone also reduced GSH levels (5.21 ± 0.13; *p* < 0.05), while the lowest GSH levels were observed in siSOX11 + BA cells (2.39 ± 0.16; *p* < 0.001 vs. Scrambled + BA), reflecting exacerbated oxidative stress.

Similarly, Fe²⁺ levels were significantly elevated in response to BA treatment (128 ± 5.14 vs. 61.47 ± 6.19 in controls; *p* < 0.001; Fig. 6E). *SOX11* silencing alone increased Fe²⁺ accumulation (92.26 ± 7.11; *p* < 0.05), whereas the combination of siSOX11 and BA led to the highest Fe²⁺ levels (215.73 ± 12.18; *p* < 0.001 vs. Scrambled + BA).

## Discussion

EC represents a highly aggressive gynecological malignancy with particularly poor prognoses for advanced-stage patients, characterized by elevated mortality risk and diminished quality of life [25]. Current National Comprehensive Cancer Network guidelines prioritize surgical resection with adjuvant chemo-radiotherapy as primary treatment [26], yet therapeutic resistance frequently manifests through metastasis and recurrence. Consequently, elucidating EC pathogenesis and developing novel diagnostic and therapeutic approaches remain imperative. Ferroptosis—an iron-dependent cell death modality distinguished by unique regulatory mechanisms—has emerged as a significant contributor to tumor progression across malignancies [27]. In EC specifically, prior evidence indicates that *SLC7A11* downregulation stimulates ferroptotic death [28], whereas epigenetic modifications elevating *SLC7A11* expression confer ferroptosis resistance and promote proliferation [29]. Collectively, these findings position ferroptosis induction as a promising therapeutic strategy. In this study, we specifically quantified the cytotoxic impact of BA in Ishikawa cells, revealing potent ferroptosis-mediated cell death. To our knowledge, this study provides preliminary experimental evidence suggesting that SOX11 signaling may regulate ferroptosis in EC cells, with BA may exert its effects, at least in part, through modulation of the SOX11/GPX4/SLC7A11 axis to induce cell death. Our findings establish that BA-triggered cytotoxicity directly correlates with its targeted downregulation of this ferroptosis-controlling pathway in Ishikawa cells.

Beyond suppressing proliferative signaling, ferroptosis induction represents a critical therapeutic mechanism in oncology [13]. Boron compounds exhibit multifaceted anticancer properties: in prostate cancer, they downregulate the cell cycle and disrupt Ca²⁺ signaling [21]. Additionally, borax induces caspase-3-dependent apoptosis in glioblastoma at low concentrations [30]. While BA suppresses breast cancer proliferation without altering p53, Bcl-2, cytochrome c, or caspase-3 activity [31], it conversely enhances endoplasmic reticulum stress while reducing viability and migration in hepatocellular carcinoma [23]. Similarly, BA impedes proliferation, invasion, migration, and clonogenicity in ovarian cancer while inducing oxidative stress and apoptosis [32]. Extending these observations to EC, a previous work demonstrated BA’s dose-dependent reduction in cell viability (IC₅₀ = 40 mM), accompanied by elevated apoptotic markers [24]. In contrast, our study revealed an IC₅₀ of 2.6 mM in Ishikawa cells after 24 h of BA treatment. This notable discrepancy may be attributed to differences in cell culture conditions (e.g., serum concentration, passage number), assay protocols (e.g., MTT incubation time, cell seeding density), and possible inter-laboratory variations in Ishikawa cell line characteristics. Furthermore, our use of a broader concentration range (100 µM–25.6 mM) and precise 24 h and 48 h exposure windows may have enhanced detection sensitivity for BA-induced cytotoxicity. Supporting this view, Paties Montagner et al. emphasized that boron compound efficacy is highly dependent on experimental parameters, including cell passage and redox status [20]. Ishikawa cells demonstrated greater sensitivity (IC₅₀ = 2.6 mM) versus SHT290 cells (IC₅₀ = 15.5 mM), representing an approximately 7-fold difference. BrdU incorporation assays corroborated this differential response, revealing substantially impaired proliferation in Ishikawa cells relative to SHT290 counterparts. Collectively, these findings highlight BA’s preferential cytotoxicity toward EC over normal cells and its cell line-specific potency. The observed variability in effective boron concentrations across studies likely reflects compound-specific properties and cellular passage heterogeneity.

The SOX transcription factor family constitutes essential regulators governing fundamental biological processes, including development, tissue homeostasis, regeneration, and cellular reprogramming [36]. Within this family, SOX11 has emerged as a candidate oncogenic driver, primarily due to its pronounced overexpression in mantle cell lymphoma [33]. Nevertheless, the expression pattern and functional significance of SOX11 exhibit considerable heterogeneity across diverse malignancies. Elevated *SOX11* expression is documented in medulloblastoma [34], whereas its transcript levels are markedly diminished in prostate carcinoma relative to benign prostatic hyperplasia [35]. In epithelial ovarian cancer, heightened *SOX11* mRNA expression correlates with enhanced disease-specific and recurrence-free survival outcomes [17]. Conversely, *SOX11* overexpression is a consistent feature observed across all subtypes of malignant breast tumors [36]. Notably, nuclear localization of SOX11 in breast cancer correlates with favorable clinicopathological indicators, encompassing lower tumor grade, absence of lymph node metastasis, reduced primary tumor dimensions, and prolonged overall survival [10]. In contrast, investigations focused on lymph-node-negative breast cancer associate *SOX11* expression with adverse clinical outcomes, including elevated risk of distant metastasis and diminished overall survival [37]. Similarly, EC tissues exhibit increased *SOX11* expression compared to adjacent non-malignant tissues, correlating with reduced disease-free and overall survival rates [38]. Parallel evidence implicates SOX4, whose expression is epigenetically augmented in EC through miR-129-2 downregulation, as an oncogene contributing to EC pathogenesis. Prior research established that SOX4 promotes ferroptosis in models of calcium oxalate crystal-induced kidney injury by facilitating enhancer of zeste homolog 2-mediated transcriptional repression of SLC7A11 [39]. Zhu and Xu subsequently provided seminal evidence demonstrating that SOX4-mediated inhibition of ferroptosis facilitates EC progression [40]. Despite these insights, the potential relationship between SOX11 and ferroptosis within EC, the influence of BA, and the mechanistic underpinnings governing this association remained undefined. This investigation unveils novel findings establishing that BA-mediated induction of ferroptosis suppresses EC progression via the SOX11/GPX4/SLC7A11 signaling cascade. Our experimental data further propose SOX11 as a viable molecular target for ferroptosis-directed therapeutic strategies in EC. Mechanistic analyses reveal that BA inhibits tumor progression through the transcriptional modulation of *SOX11*,* GPX4*, and *SLC7A11*. Specifically, SOX11 functions as a transcriptional activator of *GPX4* and *SLC7A11*, consequently augmenting cellular viability in EC. Substantiating this mechanism, BA treatment of Ishikawa cells resulted in a significant decline in *SOX11* levels, accompanied by concomitant reductions in *GPX4* and *SLC7A11* expression; this suppressive effect was effectively abrogated by pre-treatment with the ferroptosis inhibitor Fer-1. The functional silencing of SOX11 provides critical mechanistic insight into its role in regulating ferroptosis in EC cells. Our findings demonstrate that *SOX11* knockdown alone partially recapitulates the effects of BA, including suppression of *GPX4* and *SLC7A11* expression and induction of oxidative stress markers. More importantly, the combination of SOX11 depletion and BA treatment resulted in a synergistic enhancement of ferroptotic features, characterized by increased lipid peroxidation and iron accumulation alongside marked depletion of intracellular GSH. These results strongly support the notion that SOX11 acts as a positive regulator of ferroptosis defense mechanisms, primarily through maintaining GPX4/SLC7A11-mediated redox homeostasis. Consequently, BA-induced ferroptosis appears to be, at least in part, mediated through transcriptional suppression of *SOX11*. This functional validation not only strengthens the causal relationship between SOX11 downregulation and ferroptosis induction but also highlights SOX11 as a potential therapeutic target for sensitizing EC cells to ferroptosis-based treatment strategies.

This study establishes that SOX11 functions as a critical regulator in EC cells, exerting a dual effect: its downregulation suppresses cellular proliferation and promotes ferroptotic cell death. Mechanistically, SOX11 acts as a transcriptional controller, directly governing the expression of key ferroptosis regulators, GPX4 and SLC7A11. Notably, our findings indicate that BA likely induces ferroptosis and inhibits proliferation in EC cells primarily through modulating this SOX11/GPX4/SLC7A11 axis. Functional analyses robustly demonstrate that BA-mediated manipulation of SOX11, and its consequent regulation of the GPX4/SLC7A11 signaling pathway, plays a pivotal role in driving these ferroptotic and anti-proliferative outcomes. Collectively, these results suggest that BA warrants further in vivo and translational evaluation for possible application in EC treatment. Furthermore, the research uncovers a novel SOX11-dependent regulatory mechanism underpinning ferroptosis in EC, offering fresh perspectives for therapeutic intervention. It specifically provides a strong theoretical foundation for targeting the SOX11/GPX4/SLC7A11 axis in clinical EC management. Nevertheless, several limitations of the present study should be acknowledged. First, all experiments were conducted exclusively in vitro using two cell lines (Ishikawa, SHT290). While these are well-characterized models, they do not capture the tumor microenvironment, immune interactions, or three-dimensional tissue architecture. Moreover, additional EC cell lines and primary patient-derived samples are needed to confirm the generalizability of our findings. Second, the BA concentrations used (active at 2.6 mM) are considerably higher than physiological plasma boron levels (10–20 µM), limiting direct clinical extrapolation; however, such concentrations are commonly used in preclinical in vitro studies to establish mechanisms. Third, although Fer-1 reversed BA effects, we did not employ other ferroptosis inhibitors (e.g., Liproxstatin-1) or genetic rescue experiments (e.g., *GPX4* overexpression) to further confirm specificity. Fourth, while our siRNA experiments support a functional role for SOX11, we did not perform promoter reporter or chromatin immunoprecipitation assays to verify that SOX11 directly transactivates GPX4 and SLC7A11. Fifth, we focused on GPX4 and SLC7A11, but SOX11 may regulate other ferroptosis-related or cell death genes. Sixth, off-target effects of BA and its in vivo pharmacokinetics remain unexplored. Finally, the statistical comparisons were based on seven independent experiments, but potential batch effects and inter-assay variability were not systematically analyzed. Despite these limitations, our findings provide a rationale for further in vivo and mechanistic studies.

## Supplementary Information

Below is the link to the electronic supplementary material.

Supplementary figure 1 (PNG 1.01 MB)

Supplementary Material 1 (TIF 4.76 MB)

## Data Availability

No datasets were generated or analysed during the current study.
